# Red fluorescence of the triplefin *Tripterygion delaisi* is increasingly visible against background light with increasing depth

**DOI:** 10.1098/rsos.161009

**Published:** 2017-03-22

**Authors:** Pierre-Paul Bitton, Ulrike K. Harant, Roland Fritsch, Connor M. Champ, Shelby E. Temple, Nico K. Michiels

**Affiliations:** 1 Animal Evolutionary Ecology, Institute of Evolution and Ecology, Department of Biology, Faculty of Science, University of Tübingen, 72076 Tübingen, Germany; 2 School of Biological Sciences, University of Bristol, Bristol BS8 1TQ, UK

**Keywords:** visual communication, visual model, photoreceptor, fluorescence, intraspecific signal

## Abstract

The light environment in water bodies changes with depth due to the absorption of short and long wavelengths. Below 10 m depth, red wavelengths are almost completely absent rendering any red-reflecting animal dark and achromatic. However, fluorescence may produce red coloration even when red light is not available for reflection. A large number of marine taxa including over 270 fish species are known to produce red fluorescence, yet it is unclear under which natural light environment fluorescence contributes perceptively to their colours. To address this question we: (i) characterized the visual system of *Tripterygion delaisi,* which possesses fluorescent irides, (ii) separated the colour of the irides into its reflectance and fluorescence components and (iii) combined these data with field measurements of the ambient light environment to calculate depth-dependent perceptual chromatic and achromatic contrasts using visual modelling. We found that triplefins have cones with at least three different spectral sensitivities, including differences between the two members of the double cones, giving them the potential for trichromatic colour vision. We also show that fluorescence contributes increasingly to the radiance of the irides with increasing depth. Our results support the potential functionality of red fluorescence, including communicative roles such as species and sex identity, and non-communicative roles such as camouflage.

## 1. Background

Light environments change predictably with depth in the water column due to the selective attenuation of short and long wavelength irradiance by the aquatic medium [1,2]. Whereas, in clear marine waters, the ambient light at the surface (0–2 m) is nearly identical to that found on land in the visible range, wavelengths in the range of 600–700 nm (perceived as red) and those below 400 nm (in the UV range) are almost entirely lacking at more than 20 m depth [3–6]. In general, fishes are spectrally tuned to their light environment, with species found at greater depth having blue-shifted photoreceptor sensitivities [7–9]. Even fish species that occur in greater abundance near the water surface (above 10 m) are not particularly sensitive to light in the red end of the spectrum [7]. Indeed, the peak absorption of the photoreceptor most sensitive to long wavelengths averages around 520 nm in marine fish [7]; in comparison, the average peak absorption of the avian long-wavelength sensitive cones is 565 nm [10].

One of the consequences of long-wavelength attenuation in water is that materials that reflect red lose this component of the colour and appear dark grey with increasing depth [11]. This is why species of brightly coloured red and yellow reef fish appear duller in deeper waters, even when observed from a short distance, whereas blue and green coloured fishes still appear relatively bright. The presence of red coloration below 20 m, however, is not uncommon. Red fluorescence is ubiquitous in marine ecosystems and its production has been confirmed in various taxa (sponges: Porifera [12]; corals: Cnidaria [13–16]; arthropods: Arthropoda [17]; echinoderms: Echinodermata [12]; fish: Chordata [12,18]). In reef fishes alone, red fluorescence has been identified in at least 270 species, making up more than 40% of the species surveyed [5,18]. In contrast with bioluminescence, which consists of the production of light by biological material [19], fluorescence is the conversion of short-wavelength light into longer-wavelength light [11]. Therefore, the radiance of fluorescent surfaces is due to the combination of their reflective properties (reflectance) and of their fluorescent properties (fluorescence); we use the term ‘relative radiance’ to describe the combination of light reflected and fluoresced as a proportion of the ambient light field. Species surveyed to date show that the red fluorescence excitation peaks in fishes are found below 550 nm and that peak emission wavelengths range from 580 nm to over 700 nm [5,20–22]. Since photons of wavelengths below 550 nm are relatively abundant below 10 m, fluorescence, in combination with sufficiently long-wavelength spectral sensitivity, has the potential to generate perceivable red coloration in fishes starting at these depths. However, the contribution of fluorescence to complete signals (reflectance and fluorescence) at different depths has only been addressed in corals [15,23] and mantis shrimps [24].

Previous studies on red fluorescence in fishes have described its presence or absence and inferred possible functions depending on the location and intensity of the fluorescing patches [5,20–22]. Red fluorescence could contribute to signals involved in male–male agonistic interactions [20], species recognition [24], prey detection [5,25], camouflage [5,18] and mate choice [5,20,22]. In species that are not sensitive to red wavelengths, red fluorescence could offer protection against ultraviolet irradiance near the water surface, but, in contrast with corals [26], there is no evidence for this hypothesis in fish [22]. Furthermore, we know from laboratory experiments that even if fishes have low sensitivity to red wavelengths, a few species can perceive their own fluorescence [20,27]. These studies have given us important insight into the potential role of red fluorescence, yet it is unclear how much fluorescence contributes to animal signals under natural light environments.

In this study, we used visual models to better understand under which natural conditions red fluorescence produced by the irides of the triplefin *Tripterygion delaisi* could contribute to the chromatic and achromatic component of their relative radiance. To achieve this, we characterized the visual system of the triplefin, separated the colours of the irides into their reflectance and fluorescence components, and combined these data with field measures of ambient light spectra from triplefin habitats. We predicted that red fluorescence generates stronger contrast in the stenospectral zone (at depths where red wavelengths are limited [22]), and that fluorescence contributes increasingly to the perception of the signal with increasing depth. Determining how the relative radiance of fluorescent colours change in different environments will allow a better understanding of the visual ecology of fluorescence and provide further insight into the functions of fluorescence.

## 2. Material and methods

### 2.1. Model species and general methods

**Figure 1.**
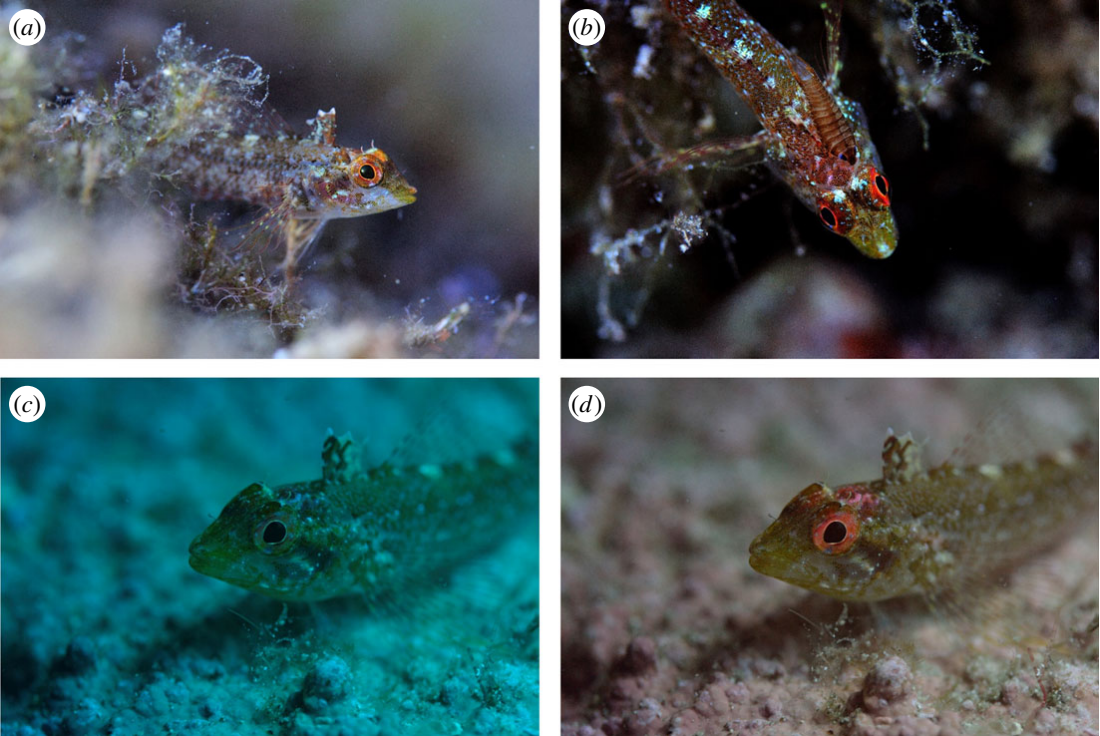
*Tripterygion delaisi* in its natural environment at 5 m (*a,b*) and at 20 m depth (*c,d*). All images were taken with natural lighting, but (*b*) was taken with the camera fitted with a long-pass filter, which emphasizes the red coloration of the head and irides; (*a,b,d*) were taken with a manual white balance, whereas (*c*) was taken with an automatic white balance. Photo credits: Nico K. Michiels.

*Tripterygion delaisi* (Family: Tripterygiidae; figure 1) is a small benthic fish species widely distributed across the northeastern Atlantic Ocean and Mediterranean Sea from a depth of 3–50 m. It forages mainly on small arthropods [28,29], which it hunts using a saltatory feeding behaviour on vegetated rocky substrates. Its general movements are limited and individuals will often freeze when approached, relying on their cryptic coloration and patterning to avoid detection. It has red-brown irides that fluoresce red under blue-green light, and a previous study on *T. delaisi* has demonstrated that it is capable of perceiving red colours similar to its own fluorescence [27]. We focus on the irides because, except for a small area on the top of the head, they are the only fluorescing body parts in this species. The fluorescence radiance increases with depth in the field [22] in response to the reduced ambient brightness rather than differences in the spectral composition [30]. These differences in fluorescence radiance are due to the movement of melanosomes within melanophores, which can obscure the guanine crystals responsible for the production of fluorescence [31,32]. In the laboratory, we characterized aspects of the visual system of *T. delaisi* by measuring: the transmission of the ocular media using spectrophotometry, the peak sensitivity of the retinal photoreceptors using microspectrophotometry (MSP) and the density of photoreceptors by mapping the retinal topography. We also objectively quantified the reflective and fluorescent properties of the triplefin irides using a spectrofluorometer with monochromators. In the field, we collected ambient irradiance measurements at different depths. We combined these data to determine if the fluorescent properties of the irides are sufficient to increase the conspicuousness of individuals when viewed by conspecifics. Using the receptor-noise model of colour vision [33], we modelled, under different light environments, the chromatic and achromatic contrasts between: (i) the iris with and without fluorescence; (ii) the iris without fluorescence (reflectance only) against an achromatic background; and (iii) the fluorescing iris (relative radiance) against an achromatic background. All data are available on Dryad [34].

### 2.2. Fish capture and post-capture care

Field data were collected between 2014 and 2015 at the Station de Recherches Sous-marines et Océanographiques de Calvi (STARESO), Corsica, France. Fieldwork took place from the beginning of June until early July in two successive years. Fish were caught while scuba-diving between 5 and 20 m using hand nets and 50 ml perforated falcon tubes (to permit water exchange during the dive). During the field trip, fish were kept in the field station in a 50 l flow-through tank at ambient water temperature, until they were transferred to aquarium facilities at the Eberhard Karls University of Tübingen, Germany. In these facilities, individuals were kept separately in blue LED-illuminated 15 l flow-through tanks (18°C, salinity 34‰, pH 8.2, 12 L : 12 D cycle, fed once per day) in a 600 l water-filtering system.

### 2.3. Light environment

Radiance measurements were taken with a spectroradiometer (SpectraScan PR-740, Photo Research, New York, NY, USA) equipped with the MS-75 standard lens fitted with an SL-0.5× add-on macro lens, and encased in a custom-made underwater housing (BS Kinetics, Achern, Germany). Because the integration time of the spectroradiometer is mainly set at depth by the abundant blue-range wavelengths, the signal-to-noise ratio in the long wavelengths was poor. To correct for this, we equipped the spectroradiometer with a Double CT Orange filter (Filter #287, Lee Filters, Hampshire, UK). The device was set up and calibrated for radiance measurements (W sr^−1^ m^−2^ nm^−1^) in the 380–780 nm range, summarized in 1 nm bins at a 1 nm resolution using a bandwidth of 8 nm. Ambient irradiance was quantified by measuring a diffuse white standard (PTFE) angled at 45° from normal to the surface. This measurement geometry captured the downwelling and side-welling light field components that will most often strike the iris of triplefins on the substrate. Three measurements were obtained every 2 m from a depth of 6 to 36 m, and the averages at each depth used for analyses. While measuring, the diver operating the spectrophotometer held their breath to avoid any potential confound due to ascending air bubbles. A compass, a level indicator and an electronic depth gauge were mounted on top of the housing for accurate positioning. All data were then corrected for the spectral transmission of the underwater housing's port, the macro lens and orange filter. Radiance measurements (W sr^−1^ m^−2^ nm^−1^) were transformed into photon irradiance (photons s^−1^ m^−2^ nm^−1^) by multiplication with *π* × wavelength × 5.05 × 10^15^ at each wavelength [11].

### 2.4. Transmission properties of ocular media

Eight individuals were sacrificed; the dermal cornea, scleral cornea and lens of each eye were dissected out with eye scissors and placed in a small Petri dish filled with enough saline to just cover each part (saline composition in mM: NaCl 125.3, KCl 2.7, CaCl_2_ 1.8, MgCl_2_ 1.8, d(+)-glucose 5.6, Tris–HCl 5.0, pH 7.2). The transmission properties of the structures were measured by means of a spectroradiometer (SpectraScan PR-670, Photo Research) attached to a Leica stereomicroscope (Leica MZ16F, Leica Mikrosysteme Vertrieb GmbH, Wetzlar, Germany) with a bright field base illuminated using a cold light source (Leica KL 2500, Leica Mikrosysteme Vertrieb GmbH). For each structure of every eye, we took three radiance measurements of the background light as seen through the structure (plus Petri dish and saline) and one of the background without the structure (but including the Petri dish and saline). Transmission was then determined as the mean of the three measurements of the structure divided by the background reference and expressed as a proportion at each nanometre.

### 2.5. Photoreceptor sensitivity

Nine fish were dark-adapted for at least 1 h before being killed by overdose (eugenol 100 mg l^−1^) followed by cervical transection under dim red light. We prepared the retinal tissue for MSP as detailed in Hart [35] and Temple *et al.* [36]. In short, we sampled small pieces (1–2 mm^2^) of the retina from five locations (central, dorsal, ventral, temporal and nasal periphery), orienting the retina using the falciform process. The absorbance spectra of the photoreceptors were recorded from the outer segments using the equipment and procedures described in Govardovskii *et al.* [37]. The measurements were obtained between 300 and 800 nm with the incident light lateral to the outer segment and polarized perpendicularly to its longitudinal axis. Each pre-bleach absorbance spectrum was produced by subtracting a scan of a tissue-free area of the preparation from a non-bleached photoreceptor scan. Similarly, each post-bleach absorbance spectrum was produced by subtracting a scan of tissue-free area from a photoreceptor that was bleached for 1 min with full-spectrum light. Final absorbance profiles were calculated by subtracting the post-bleach spectra from the pre-bleach spectra, and analysed using procedures described in Hart [38]. We compared the *λ*_max_ values of photoreceptors from different retinal areas using one-way ANOVA, and controlled for a false discovery rate [39] by adjusting the *p*-values for multiple comparisons (four photopigments). All statistical analyses were conducted using R [40].

### 2.6. Photoreceptor densities

The relative density of the different photoreceptor types is an important visual model parameter because it has a large influence on the accuracy of perceived contrast [41,42]. To determine the ratio of single to double cones in *T. delaisi*, we produced retinal wholemounts of the eyes of four individual*s*. We followed a slightly modified protocol for receptor cells presented in Coimbra *et al.* [43] and give only an overview of the procedures here. The fish were euthanized by immersion in seawater with 1 g l^−1^ tricaine mesylate (MS-222), buffered to pH 8.2, and death was ensured via spinal cord cut. The light-adapted eyes were excised immediately and their corneas, iris, lens and vitreous body removed while kept in Ringer solution. After immersion–fixation of the remaining eyecup with 4% paraformaldehyde in 0.1 M phosphate buffer for 25 min, four to five radial cuts were applied to facilitate removing the sclera and choroid, as well as flattening the retina. The retinal pigment epithelium was bleached with 12% hydrogen peroxide in 0.9% phosphate-buffered saline for 5 h at room temperature. Finally, the retina was transferred to an uncoated slide, flattened and mounted in 80% glycerol, and the coverslip edges sealed with nail varnish. After letting the tissue clear for at least 24 h, the receptor mosaic pattern was inspected under a DIC microscope (Leica DM5000B, Leica Mikrosysteme Vertrieb GmbH).

### 2.7. Reflective and fluorescent properties

We separated the reflectance and fluorescence component of the triplefin iris with a Quantamaster QM 40 (Photon Technology International, Bensheim, Germany) connected to a Leica stereomicroscope (Leica MZ16F, Leica Mikrosysteme Vertrieb GmbH) using liquid light guides (LLG 380, Photon Technology International, New Jersey, USA). The samples were immersed in a seawater Ringer solution with K^+^ elevated solution (mM: NaCl 78, KCl 50, CaCl_2_ 1.8, MgCl_2_ 1.8, d-(+)-glucose 5.6, Tris–HCl 5.0, pH 7.2). Elevated levels of potassium result in melanosome aggregation, thus exposing the fluorescent surface, allowing us to capture the potential fluorescence of the sample [30,32]. We first characterized a polytetrafluoroethylene (PTFE) white diffuse standard (Berghof Fluoroplastic Technology GmbH, Eningen, Germany) by sequentially shining monochromatic light (range 400–700 nm in 5 nm intervals for three fish and in a 4 nm interval for one fish) onto the sample, and collecting the emitted light across the entire visible range (range 380–720 nm in 5 nm intervals for three fish and 2 nm intervals for one fish) for every excitation interval. We characterized the irides (eight eyes from four fish) of triplefins using the same sequence of measurements, and all spectra were interpolated to 1 nm intervals. Following acquisition of these data, we corrected the spectral curve of each scan for the wavelength dependency of the photon counter and light absorbance by the equipment between the sample and the photon counter (liquid light guides, collimating lenses and microscope) with a calibration function derived from measurements made using a spectroradiometer (SpectraScan PR-740, Photo Research). We calculated the reflectance component of the signal by comparing the difference in emitted photons between the white standard and the samples at the emission wavelength that coincided with the current excitation wavelength (inelastic scattering). This implicitly assumes that fluorescent emission is negligible whenever the excitation and emission intervals are identical. To calculate the fluorescence component of the signal, we first corrected the spectral curve of the sample emission scan at each excitation interval for the incomplete quench of the monochromator by subtracting the product of the sample reflectance per nm and the white standard emission. A complete fluorescence probability function was then extracted by (i) calculating the difference in photons reflected between the white standard and sample (all photons absorbed) and (ii) calculating the proportion of these non-reflected photons and the ones re-emitted at longer wavelengths (elastic scattering) [24]. This is a conservative measure of fluorescence efficiency since we measured natural tissue and photons were not just absorbed by fluorescent guanine crystals. This resulted in a complete excitation by emission wavelength matrix with values for fluorescence efficiency (proportion) for each excitation value at all longer emission wavelengths.

### 2.8. Visual models of conspecific detection

The relative radiance of the eyes of triplefins was produced by multiplying the reflectance vector by the downwelling irradiance at various depths and adding the contribution of fluorescence obtained by applying the ambient light vector to the fluorescence efficiency matrix. To determine how the fluorescent component of the red iris signal contributes to the conspicuousness of triplefins to conspecifics at different depths, we modelled the chromatic and achromatic contrasts between 6 and 36 m of (i) the iris with fluorescence and without (reflectance only), (ii) the iris reflectance (no fluorescence) against achromatic backgrounds and (iii) the fluorescing iris (relative radiance) against achromatic backgrounds. We produced backgrounds of different brightness using achromatic spectra (equal intensity across all wavelengths from 400 to 700 nm) with varying mean brightness because many marine substrates are achromatic [44,45] (mean brightness = 5% to 50% in 2.5% intervals). We calculated the chromatic and achromatic contrasts in just-noticeable differences (JNDs), where scores above 1.0 indicate that the colours are distinguishable from one another, using Vorobyev and Osorio's receptor-noise model [33]. We used the visual system measurements obtained from this study to inform the model, and set the Weber fraction at 0.05 for the long-wavelength sensitive cone [33]. We calculated achromatic contrasts for the double cones, also assuming a Weber fraction of 0.05. We assumed that most interactions between individuals occur at distances of less than 10 cm for which light scattering would be negligible [44]; therefore we did not include an attenuation coefficient and distance parameter in any model. We included the measured irradiance, which combines the downwelling and side-welling light fields, at the appropriate depth as the adapted background (von Kries correction) [44,46]. We compared the signals with and without the contribution of fluorescence at depths ranging from 6 to 36 m, in 2 m intervals. We used the package pavo [47] for the R programming language [40] to produce the photoreceptor sensitivity curves for the models and to calculate the chromatic and achromatic contrasts.

## 3. Results

### 3.1. Visual system

*Tripterygion delaisi* possesses a dermal (outer) and a scleral (inner) cornea. Both corneas are characterized by transmissions of more than 90% across the spectral range between 400 nm and 700 nm. We found no iridescent structures in the corneas, as has been reported in other fishes [48,49]. The lens was also highly transparent, but with distinct shortwave absorption characterized by a reduction to 50% transmission at 416 nm, and only 10% transmission at 400 nm (electronic supplementary material, figure S1). The retina of *T. delaisi* is dominated by cones, which are found throughout (details to be published elsewhere). Rods occur in the entire retina, except for the fovea, with increasing numbers towards the periphery. The overall cone pattern is a regular square mosaic with a ratio of two double cones to one single cone. The fovea and surrounding area, however, feature a distinct and regular pattern with each single cone being surrounded by four double cones and no rods. Where these patterns transition from one to the other, the mosaic takes on irregular, intermediate ratios.

**Figure 2.**
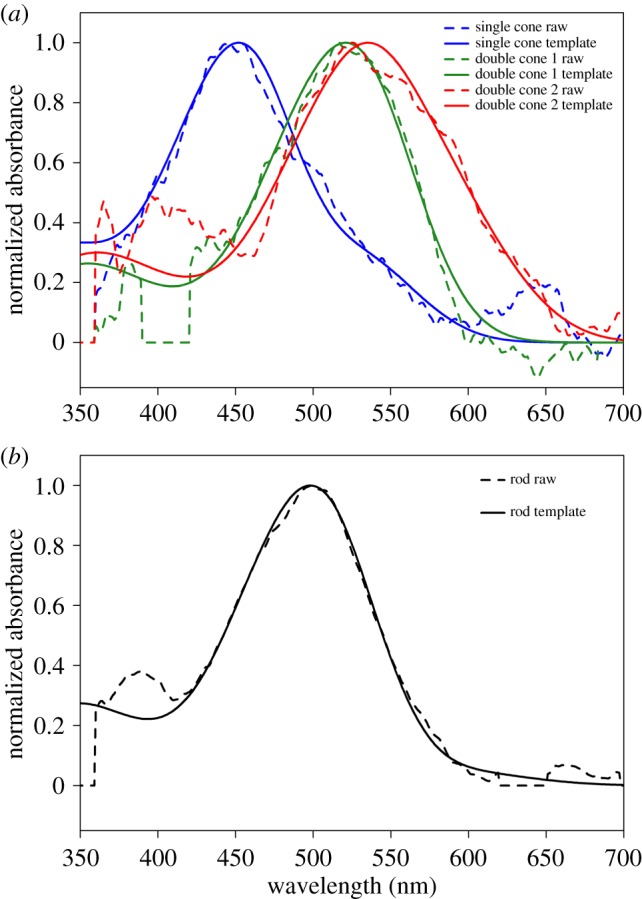
Representative photoreceptor absorbance curves (dashed lines) and predicted normalized absorbance (solid lines) of (*a*) the single and double cones and (*b*) the rods of *T. delaisi*.

**Figure 3.**
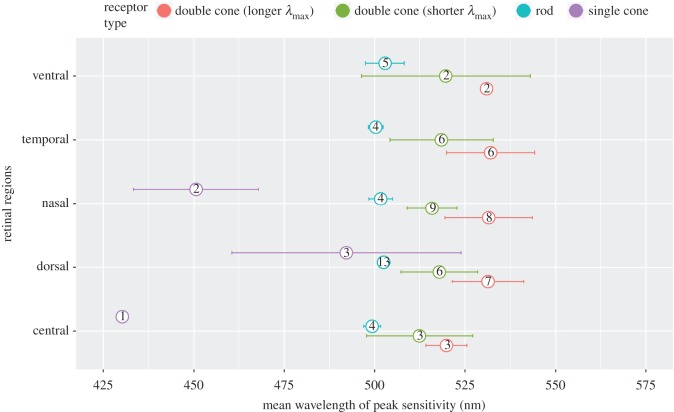
Mean wavelength of maximum sensitivity of photoreceptors from different retinal regions in *T. delaisi*. Only paired short and long wavelength-sensitive members of double cones are included (see Results). Error bars indicate 95% CIs and values in symbols indicate sample size.

We successfully measured the spectral absorbance from 115 photoreceptors sampled from the ventral, nasal, dorsal, temporal and central locations across the retinae of nine triplefins (example measurements in figure 2). We measured 30 rods, six single cones and 79 members of double cones of which 52 were paired. The 27 double cone members that were not paired could neither be assigned to the short-wavelength sensitive nor to the long-wavelength sensitive member due to the considerable overlap in *λ*_max_ values and were therefore analysed separately from the paired double cones. The *λ*_max_ values of all photoreceptors seemed to vary slightly across the retinal regions (figure 3), but the observed differences were not statistically significant after controlling for false discovery rates (rods: mean = 501.7 ± 2.9 s.d., *F*_4,25_ = 1.42, *p*-value = 0.51; single cones: mean = 468.0 ± 28.7 s.d., *F*_2,3_ = 17.17, *p*-value = 0.09; double cone shorter *λ*_max_: mean = 516.8 ± 9.5 s.d., *F*_4,21_ = 0.26, *p*-value = 0.90; double cone longer *λ*_max_: mean = 530.2 ± 11.3 s.d., *F*_4,21_ = 0.65, *p*-value = 0.85). Because of these results, we treated *T. delaisi* as trichromatic in the models [50]. The non-paired double cone members possessed pigments with wavelength of maximum sensitivity very similar to that of the paired longer *λ*_max_ cones (mean = 530.4 ± 14.15 s.d.), which also did not differ significantly by retinal region (*F*_3,23_ = 0.56, *p*-value = 0.65).

### 3.2. Visual signal

**Figure 4.**
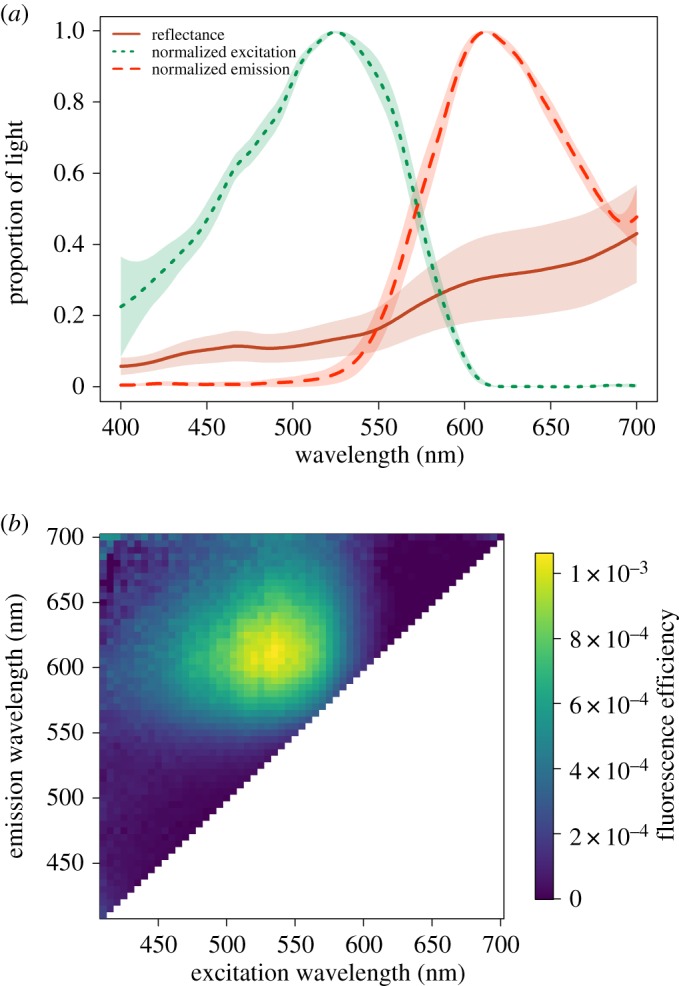
(*a*) Average (±s.d.) reflectance spectra and average (±s.d.) normalized fluorescence excitation and emission efficiency of eight *Tripterygion delaisi* irides. The colours of the curves are approximations of how they would be perceived by humans based on RGB scores of the spectral shapes. (*b*) Practical fluorescence efficiency at every 5 nm of excitation wavelength for each 5 nm of emission wavelength. Fluorescence efficiency is defined as the quotient between the number of photons emitted and photons absorbed (not reflected) and is unitless.

**Figure 5.**
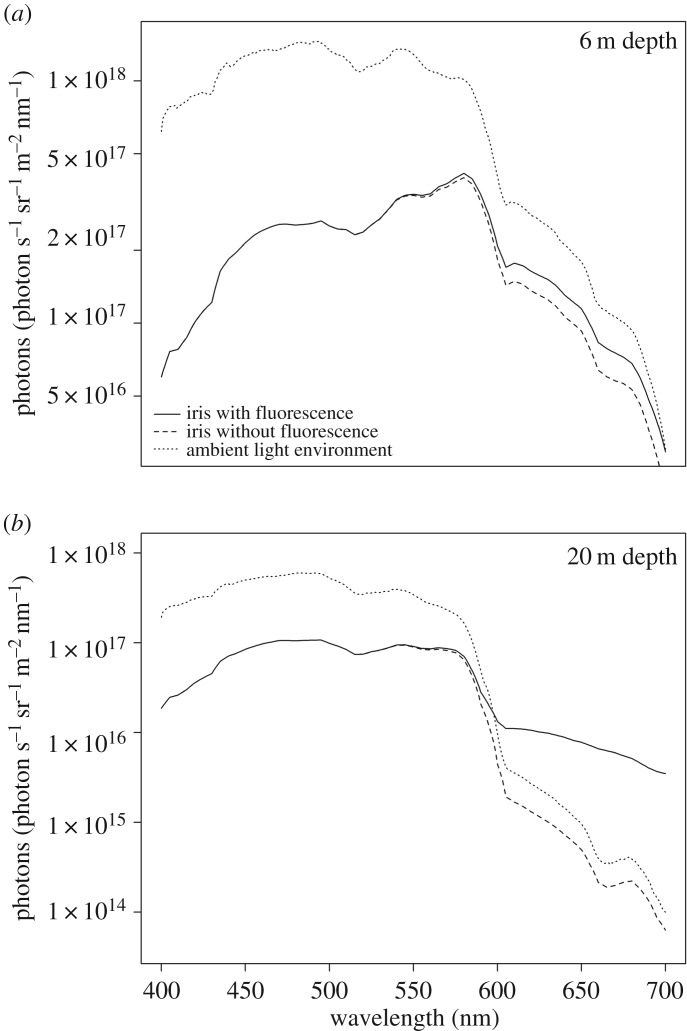
Radiance of *T. delaisi* iris with and without the contribution of fluorescence at 6 m (*a*) and 20 m (*b*), along with the respective ambient irradiance.

**Figure 6.**
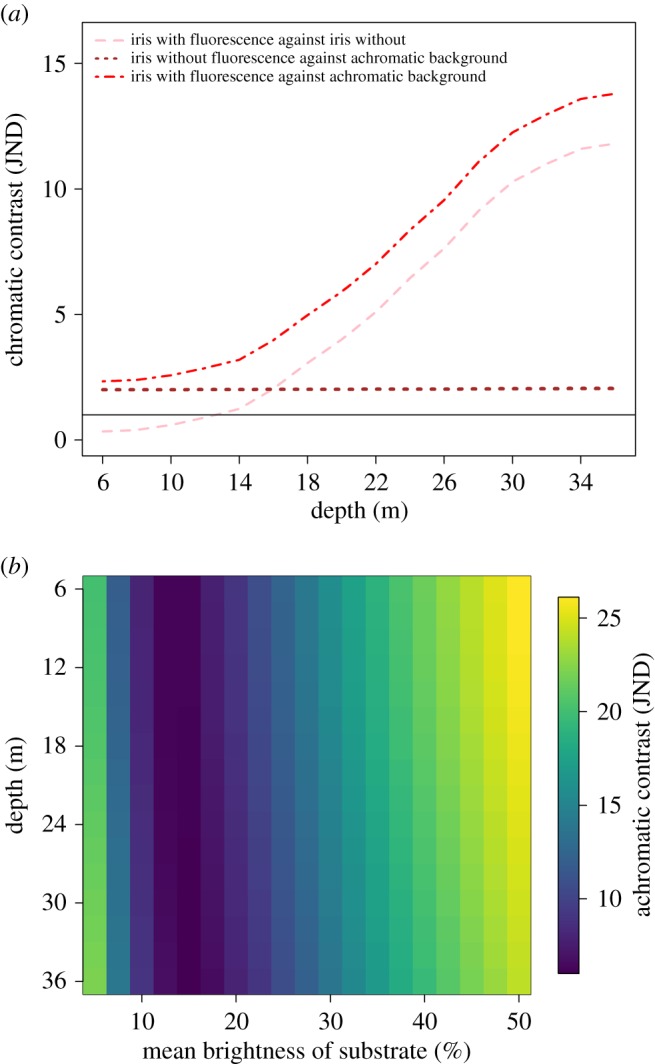
(*a*) The iris of *T. delaisi* increases in chromatic contrast with depth against an achromatic background because of red fluorescence. Solid line indicates 1 JND. (*b*) The achromatic contrast of a *T. delaisi* red-fluorescing iris against an achromatic background is influenced by depth. Against a dark background, the iris is increasingly brighter with depth (left). Against a bright background, the iris is much darker at shallow depths but reduces in contrast with greater depth (right).

Based on its spectral shape, the reflectance of the iris of *T. delaisi* (without the contribution of fluorescence) is reddish-brown (figure 4). The wavelength of peak fluorescence excitation averaged 525 nm across all eight eyes characterized with a full width at half maximum range of 452–575 nm (figure 4). The wavelength of peak fluorescence emission averaged 609 nm with a full width at half maximum range of 572–686 nm (figure 4). The practical fluorescent efficiency of the peak excitation wavelength (proportion of non-reflected photons converted to longer-wavelength photons) averaged 3.34% (range: 2.00–6.24). The contribution of red fluorescence to the visual signal of the iris varied with depth (figure 5). At 6 m (figure 5*a*), for example, the contribution of the fluorescence had little influence on the relative radiance of the irides, which never surpassed the long-wavelength irradiance available in the ambient light field. By contrast, the contribution of fluorescence to the relative radiance at 20 m was great (figure 5*b*), such that the relative radiance surpassed the long wavelength irradiance available in the ambient light field. Quantification of perceptual contrasts determined that between 6 and 12 m fluorescence did not contribute perceptively to the chromatic component of the signal (figure 6*a*). However, below 14 m the contribution of fluorescence to the chromatic component of the signal was greater than 1 just-noticeable-difference, the minimum threshold for colour discrimination (1.25 JND at 14 m), and subsequently increased with depth at an average rate of 0.53 JND m^−1^. The contrast between the iris and an achromatic background depended more on the brightness of the background than on the contribution of the fluorescence (figure 6*b*). In simulations in which the background reflectance averaged 15% or less, the iris was perceptively brighter near the surface (6 m), slightly increasing with increasing depth. When the background reflectance averaged more than 15%, the iris was perceptively darker, but less so with increasing depth.

## 4. Discussion

Over 270 species of reef fishes are known to fluoresce red but the function of these complex signals has only been tested in a handful of species [20,27]. Furthermore, although repeatedly suggested, it has never been demonstrated under which natural environmental light conditions red fluorescence is likely to contribute to visual signals. To better understand the importance of red fluorescence for the visual ecology of the triplefin *T. delaisi*, we characterized its visual system, separated the iris colour into its reflectance and fluorescence components, quantified the light environment of triplefin habitat and used these data to parametrize visual models. We found that triplefins have (i) double cones with photoreceptor sensitivities differing by an average of 14 nm, which, it may be reasonable to assume, contribute to a trichromatic colour vision system as has been reported in other marine fish species [50], (ii) fluorescent irides that convert wavelengths of light available in a wide range of depths to longer wavelengths lacking in the stenospectral zone and (iii) irides with fluorescence that contributes to the perceived chromatic contrast of the iris against an achromatic substrate [27]. Our results strongly suggest that *T. delaisi* should be capable of perceiving their own fluorescence in various natural light environments, the signalling importance increasing with depth. This is the case even if the photoreceptor sensitivity of the double cone does not appear optimized for the detection of the red fluorescence. Because this red-fluorescent radiance would be quickly absorbed through the water column, it is likely to serve a short-distance role only.

Our MSP measurements of double cones demonstrated that the short-wavelength sensitive member (516 nm) and the long-wavelength sensitive member (530 nm) are red-shifted by 25 nm and 10 nm, respectively, compared with the average for other species with a near surface distribution [7], suggesting that long-wavelength signals are important to the ecology of *T. delaisi*. Colour discrimination in the red range of the spectrum should be further possible due to the presence of double cone members with different peak sensitivities [50]. However, the difference in the peak sensitivity of the two members (average of 14 nm) is much smaller than found in the reef fish *Rhinecanthus aculeatus* (50 nm difference), the only species for which experimental evidence exists for colour discrimination through double cones [50,51]. Only behavioural tests on trained *T. delaisi* would confirm that their ability to detect their own fluorescence [27] is facilitated through trichromacy. Nonetheless, our results demonstrate that fluorescence produced by the irides of *T. delaisi* could contribute to the signal as perceived by conspecifics.

Speculation on the role of fluorescence in *T. delaisi* is hampered by the lack of basic information regarding its biology and ecology. Nonetheless, there is the potential for this fluorescent colour to be of use in intra-specific interactions in a communicative role. As with patches coloured with non-fluorescent pigments and structural colours [52,53], a fluorescent signal could be used for species recognition. In areas where *T. delaisi* inhabit areas with similar-looking species that do not possess fluorescent irides (e.g. *Parablennius zvonimiri* [28]), bright red irides would be an easily identifiable species-specific trait. It is also possible that red fluorescence contributes to sex identification. While there are differences in coloration between males and females during the breeding season (males turn bright yellow with black faces, including their irides), there is little difference between the sexes during the non-breeding season. It is possible that male and female *T. delaisi* differ in the intensity of their fluorescence; surveys of red fluorescence in reef fishes have found sexual differences in the distribution and intensity of fluorescence [12,21]. Assessment of iris fluorescence of individuals of known sex (identified during the breeding season or through examination of gonads in sacrificed fish) could help resolve any sex-related differences. Unfortunately, we did not inspect the gonads of the fish we sacrificed and cannot comment on any possible trends. Additionally, colourful secondary sexual characteristics are condition-dependent in several vertebrate taxa, and indicative of an individual's potential fitness (see [54] for examples in diverse taxa). It is possible that fluorescence plays a similar role but variation in fluorescence efficiency across individuals found at the same depth is completely unknown. Of particular interest to the signals produced by fluorescence is that all the functions described here would be depth- and, therefore, context-dependent. Furthermore, all interactions would have to take place at relatively close distances because the red coloration is quickly dulled through absorption by the water. More information pertaining to the species’ behaviour in a natural setting during both the breeding and non-breeding season would be of great value for interpreting our results.

Other non-communicative functions should also be considered. For example, it is possible that fluorescence contribution to the overall eye (and head) coloration of triplefins increases camouflage against backgrounds that we did not measure here. Several underwater algae are known to fluoresce and, in a heterogeneous environment composed of both fluorescing and non-fluorescing materials, it would be possible to reduce conspicuousness of an otherwise dark iris and pupil by producing some red coloration. The strength of this hypothesis is limited, however, because plant materials common in *T. delaisi* habitat typically fluoresce maximally at 680 nm because chlorophyll is the primary fluorescent pigment [55], and *T. delaisi* produce fluorescence that peaks at 609 nm.

We used the ambient light measured at 45° from normal to the surface, capturing both the contribution of the downwelling and side-welling light field that would usually strike the iris. This approach contrasts with most studies of underwater visual ecology by not separating the downwelling and side-welling fields; the downwelling field is typically considered the main contributor to the irradiance of a surface, while the side-welling is used as the adaptive background for colour constancy (von Kries correction). One of the implications is that our assessment of the contribution of fluorescence to the perceived contrasts calculated in this study would be underestimated. Indeed, side-welling irradiance is always much more blue wavelength-shifted compared with downwelling irradiance between 0 and 40 m [11]. Controlling for colour constancy using exclusively side-welling irradiance, fluorescence of the iris would start contributing to the signal at shallower depths and generate stronger perceived contrasts than those calculated. Further complicating matters, ambient illumination almost certainly affects discrimination thresholds but these effects have never been behaviourally tested [56]. Discrimination thresholds may be higher when the patch being evaluated matches the background [57], which would be the case in our achromatic contrast calculations below a depth of approximately 10 m.

As demonstrated by this study, red fluorescence associated with guanine–hypoxanthine crystals in the skin of fish can generate a perceivable contribution to the chromatic and achromatic component of a visual signal. The contribution to the colours produced are strongly depth-dependent and may only play a role below 12 m, at least in *T. delaisi*. Because the distribution of red fluorescence in fishes is species-, depth- and even sex-specific in many species, there are strong reasons to suggest that it has an adaptive role. A better understanding of its contribution to visual signals in other species in the context of their ecology and biology and experiments to test for specific functions will be needed to truly understand the role of red fluorescence in fish coloration.

## Supplementary Material

Figure S1 The mean transmittance of the dermal cornea, scleral cornea, and lens of the triplefin Tripterygion delaisi

## Data accessibility

Data have been archived to Dryad: http://dx.doi.org/10.5061/dryad.t7m7g [34].
