# Supplementary material for: Red fluorescence of the triplefin Tripterygion delaisi is increasingly visible against background light with increasing depth
Source: R Soc Open Sci. 2017 Mar 22;4(3):161009. doi: 10.1098/rsos.161009 (PMC5383848; doi:10.1098/rsos.161009)
Supplement: Figure S1 The mean transmittance of the dermal cornea, scleral cornea, and lens of the triplefin Tripterygion delaisi [file rsos161009supp1.docx]

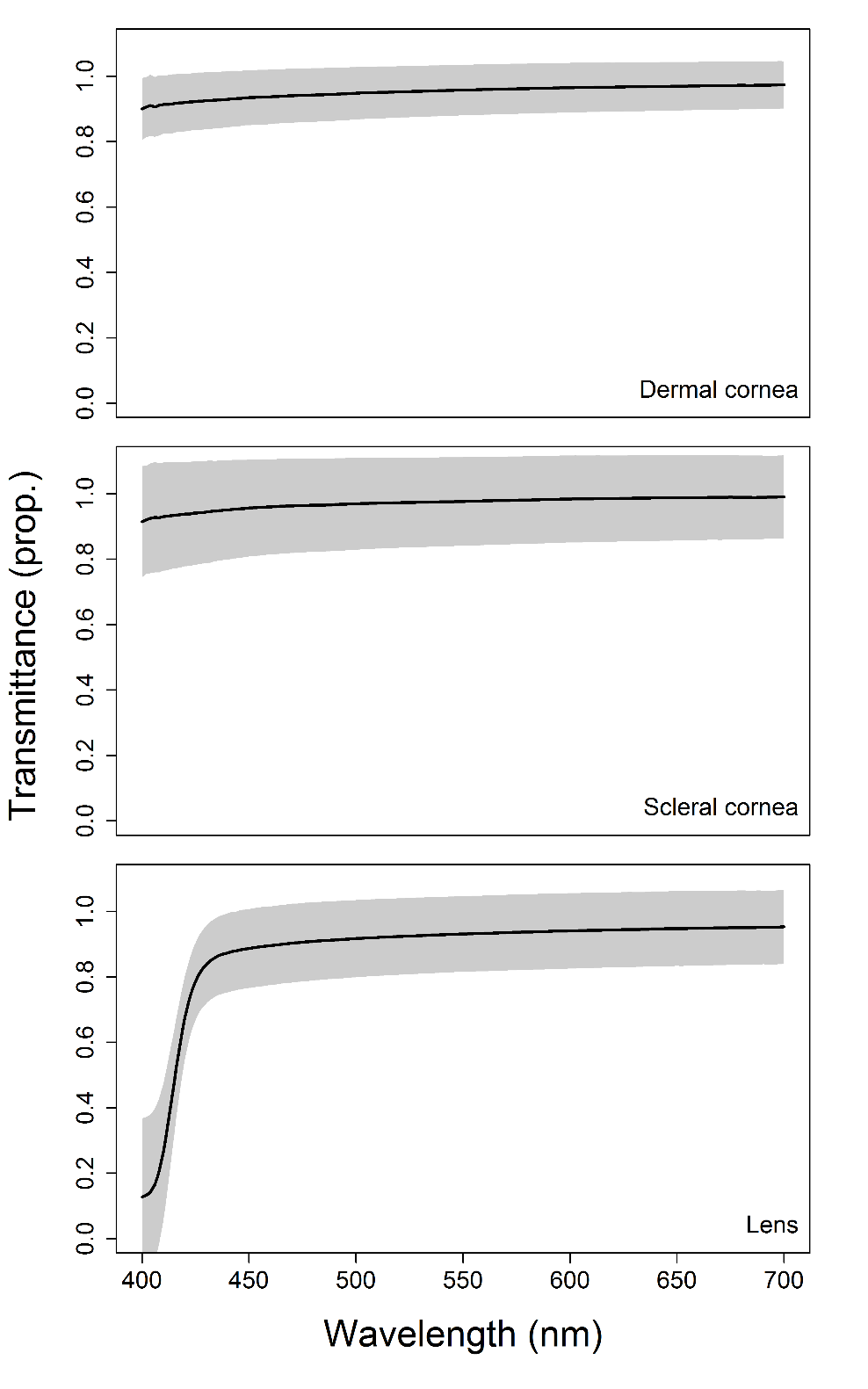


**Figure S1** Mean transmittance of the dermal cornea (top panel, n = 16), scleral cornea (middle panel, n = 16), and lens (bottom panel, n = 15) of the triplefin *Tripterygion delaisi*. Shaded area represent the standard deviation calculated at each wavelength.
